# Cosmetic products and health concerns: insights from 1149 Saudi women

**DOI:** 10.1097/JW9.0000000000000232

**Published:** 2025-12-11

**Authors:** Mohammed Nasser Asiri, Mohammed Yousof Bakhiet, Haya Abdulaziz Kisan Alzahrani, Sara Mahfoud Hassan Alghamdi, Rania A. Alghamdi, Amirah Saleh Abdullah Alzubaidi, Abdullah Faisal A. Albukhari

**Affiliations:** a Department of Surgery, Faculty of Medicine, Al-Baha University, Al-Baha, Saudi Arabia; b Faculty of Medicine, Al-Baha University, Al-Baha, Saudi Arabia; c Collage of Medicine, Umm Al-Qura University, Makkah, Saudi Arabia; d Faculty of Medicine, King Abdulaziz University, Jeddah, Saudi Arabia

**Keywords:** adverse effects, awareness, beauty, cosmetics, Saudi women

## Abstract

**Background:**

The cosmetics industry has grown significantly, driven by beauty standards and media influence. While cosmetics enhance appearance, many contain chemicals that may cause adverse effects. Understanding usage patterns and associated risks is essential for consumer safety.

**Objective:**

This study assessed cosmetic usage patterns and side effects among Saudi women, focusing on commonly used products, purchasing behaviors, and awareness.

**Methods:**

A cross-sectional survey was conducted from April 2024 to March 2025, involving 1,149 Saudi women. Data were collected via structured questionnaires on product usage, side effects, and purchasing habits. Statistical analysis examined associations between age and usage patterns.

**Results:**

The most used cosmetics were face creams (73.4%), makeup (70.2%), and hair care products (59.1%). Reported side effects included acne (76.1%), skin redness (74.3%), and hair loss (56.3%). Most participants (54.6%) bought cosmetics from supermarkets, and 72% did not read product leaflets. Younger participants (18–22 years) prioritized brand reputation and discontinued products upon experiencing side effects.

**Limitations:**

Self report, urban bias, and cross sectional design limit validity.

**Conclusion:**

The current study highlights age-related differences in cosmetic use and awareness, emphasizing the need for consumer education and stricter regulations to ensure product safety.

## Background

In today’s industrialized world, the cosmetics industry is one of the fastest-growing sectors. Cosmetics refer to products designed for application to the human body with the purpose of enhancing appearance without altering its structure or physiological functions.^1^ The global use of cosmetics has surged, largely driven by media-imposed beauty standards.^2^ Studies have detected preservatives such as parabens, carboxylic acids, alcohols, and their derivatives in various cosmetic products, including mascaras, body lotions, face creams, and leave-on formulations. These compounds have been linked to both local and systemic adverse effects, including skin irritation, rashes, blisters, and burning. Additionally, they may disrupt estrogen levels, potentially impairing female fertility, contributing to obesity, and causing hepatotoxicity and nephrotoxicity through inflammatory mechanisms.^3–10^ Furthermore, heavy metal nanoparticles—including zinc oxide (ZnO), titanium dioxide (TiO₂), aluminum oxide (Al₂O₃), and gold (Au) are widely used in cosmetics to protect the skin from ultraviolet radiation and microbial activity. However, these metals are recognized as systemic toxicants and have been associated with developmental abnormalities, cardiovascular diseases, neurological disorders, and various cancers.^11,12^

Notwithstanding these concerns, most consumers prioritize the immediate aesthetic benefits of cosmetic products over their potential long-term effects. These products are generally perceived as safe and well-tolerated.^13^

Given that Saudi women are known for their keen interest in fashion and beauty trends, numerous studies have been conducted to assess cosmetic usage patterns in this population. Most current research is constrained by limited scope or regional representation, indicating a necessity for more extensive studies throughout Saudi Arabia. Although there is an increasing occurrence of negative cosmetic effects, national-level cosmetovigilance systems are absent, emphasizing the need for assessments tailored to specific contexts.^14^ A study conducted in the Kingdom of Saudi Arabia over 3 months found that 50.6% of the 425 respondents had experienced at least one adverse reaction in the 2 years preceding the study. The most commonly reported reactions included skin redness (19%), acne (15%), and itching (13%). Skincare (25%) and hair care (29%) products were associated with a significant proportion of these adverse effects. Notably, most affected individuals (n = 181 [84.2%]) managed their symptoms by discontinuing the use of the product.^14^ Another study found that 114 of 709 female participants (16.1%) experienced adverse effects from cosmetic use. Lotions were the most frequently reported culprit (51.2%). The recorded side effects included skin redness, itching, discomfort, hair loss, eye irritation, armpit darkening, and facial pigmentation.^15^

Recent studies indicate that consumers have limited awareness of the potential adverse effects of cosmetics. Despite varying education levels, most individuals lack expertise in cosmetic products and express concerns about their use.^16^ Aladwan et al.^15^ and Manoj et al.^16^ highlighted the need for further research to assess cosmetic usage patterns. Furthermore, it has been suggested that many harmful and serious effects of cosmetics remain unreported. In addition, there is an absence of public health initiatives specifically designed for cosmetic safety that are adapted to regional needs. Given Saudi Arabia’s distinct sociocultural context and market conditions, there is a pressing need for more relevant data to inform national awareness and regulatory measures.^17^ To bridge this knowledge gap, the present study explores the usage patterns and associated side effects of cosmetics among the Saudi female population.

## Methods

### Study design, setting, and period

This online, survey-based, descriptive cross-sectional study assessed cosmetic usage habits and reported adverse effects across the Kingdom of Saudi Arabia between April 2024 and March 2025. The target population was reached through a questionnaire distributed via various social media platforms. Participants were categorized based on geographic distribution, distinguishing between Saudi women living in urban and rural areas.

### Participants

The study population consisted of adult Saudi females. Inclusion criteria required participants to be Saudi women aged 18 years or older and Arabic speakers. Non-Saudi individuals, non-Arabic speakers, and females below the specified age were excluded from the study.

### Sample size

Sample size was estimated using the following formula:


n=P(1−P)×Zα2/d2with a confidence level of95%.

n: Calculated sample size Z: The z-value for the selected level of confidence (1 − a) = 1.96

P: An estimated knowledge Q: (1 − 0.50) = 50%, that is, 0.50 D: The maximum acceptable error = 0.05 So, the calculated minimum sample size was: n = (1.96) 2 × 0.50 × 0.50 / (0.05) 2 = 385 consumers.

### Data collection tool

The survey was developed based on existing literature^13^ and the expertise of plastic surgery professors from the Faculty of Medicine at Al-Baha University. The Arabic version was distributed to the target population, with translation performed by senior medical students and validated by 3 female graduates with bachelor’s degrees in Arabic Language and Literature. The questionnaire comprised 3 main sections: consumer demographics, reported side effects, and usage patterns. Participants provided informed consent before completing the survey. The demographics section collected information on age, social status, residence, educational level, and monthly income. Additionally, cosmetic consumption habits and potential side effects were assessed through a series of 10 structured questions.

### Data analysis

Statistical analysis was conducted using SPSS version 26, with a significance level set at *P* < .05. Participant categorization was performed using descriptive statistical measures, including mean and standard deviation. Additionally, the Chi-square test was used to examine the correlation between age groups, reported adverse effects, and cosmetic consumption behavior.

### Data assurance

A preliminary test was conducted with 31 Saudi females from different regions before the main study. This pilot study was excluded from the final analysis to evaluate the questionnaire’s clarity, suitability, and validity within the study context. Based on participant feedback, the authors included a section for suggestions regarding potential modifications to ensure the questionnaire accurately reflected the target population’s perspectives. Participants confirmed the questionnaire’s clarity by responding to an open-ended question, and no further modifications were necessary before data collection began.

## Results

### Sociodemographic characteristics

Of 1,506 participants, 1,149 met the inclusion criteria. All respondents were Saudi females aged 18 years or older, with a maximum age of 54 years. The mean age of the participants was 25.3 ± 5.8 years. The majority were single (89.8%), followed by married participants (9.5%). Most respondents (82.7%) resided in urban areas. In terms of education, 54.7% had completed secondary education, while 42.6% had attained higher education. Regarding monthly income, 49.3% earned between 5,000 and 10,000 SAR, while 43.3% earned less than 5,000 SAR (Table 1).

**Table 1:** Sociodemographic characteristics of study participants

Sociodemographic characteristics		N (%)
Age	18 or more N (%)	1149 (100%)
	Mean and SD	25.3 ± 5.8
	Minimum–maximum (years)	18–54
Marital status	Single	1032 (89.8)
	Married	109 (9.5)
	Widowed	1 (0.1)
	Divorced	7 (0.6)
Residency	Town	199 (17.3)
	City	950 (82.7)
Education	Primary	1 (0.1)
	Secondary	629 (54.7)
	High level	490 (42.6)
	Postgraduate	29 (2.6)
Income	Less than 5k	498 (43.3)
	5k–10k	567 (49.3)
	More than 10 k	84 (7.3)

SD, standard deviation.

### Cosmetic products use and side effects

**Fig. 1.**
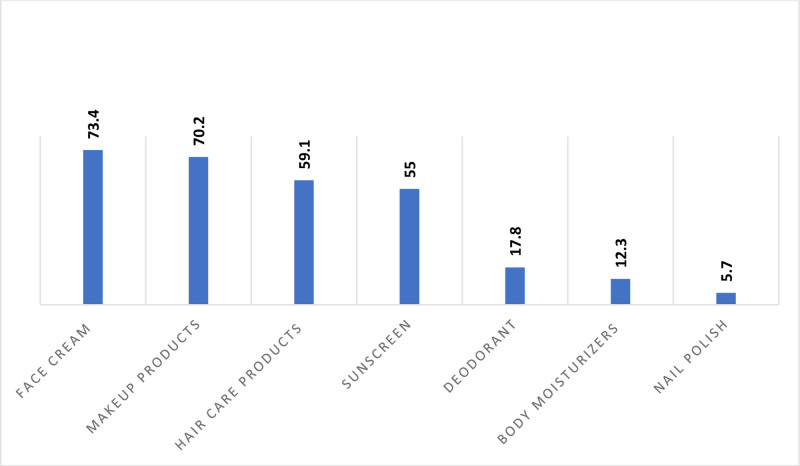
X-axis shows the different cosmetics used and y-axis shows the percentage of using these cosmetics and it shows face cream and makeup products are the most frequently used.

**Fig. 2.**
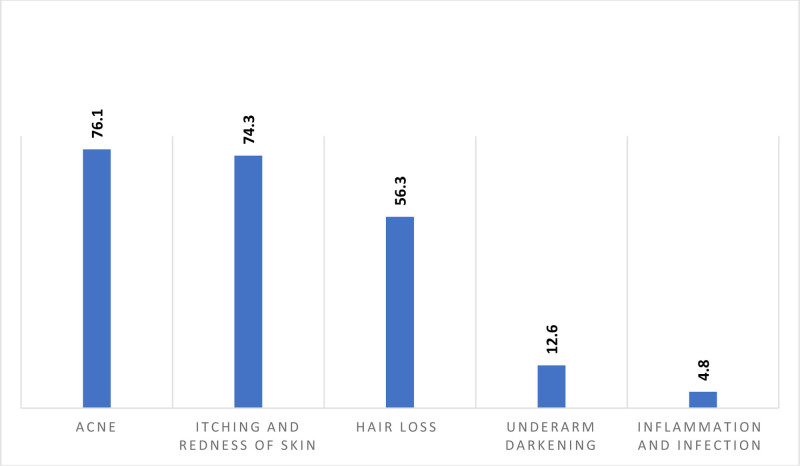
X-axis shows the different cosmetic-related adverse effects, and y-axis shows the percentage and it shows acne and itching and redness of skin are the most frequent adverse effects.

As shown in Figure 1, face creams were the most commonly used cosmetic products (73.4%), followed by makeup (70.2%) and hair care products (59.1%). The primary reasons for using cosmetics included enhancing physical appearance (66.1%), reducing skin pigmentation (59.7%), and preventing hair loss and damage (56.6%). Figure 2 illustrates the most frequently reported side effects, including acne (76.1%), skin redness and itching (74.3%), and hair loss (56.3%). Additionally, the majority of participants purchased their cosmetics from supermarkets (54.6%) (Table 2).

**Table 2:** Cosmetic product use and side effects

Product and side effects		N (%)
Type of the cosmetic product that you used and caused side effects (you can choose more than one option)	Face creams	841 (73.4)
	Hair care products	677 (59.1)
	Makeup products	804 (70.2)
	Sunscreen	629 (55)
	Deodorant	204 (17.8)
	Nail polish	65 (5.7)
	Body moisturizers	141 (12.3)
Why did you use the cosmetic (you can use more than one answer)	Enhance physical appearance	757 (66.1)
	Reduce hair loss and damage	648 (56.6)
	Skin whitening	590 (51.5)
	Reduce skin pigmentation	684 (59.7)
	Follow beauty trends	122 (10.6)
	Cover skin flaws	194 (17)
What side effects did you experience when you used this cosmetic? (you can choose more than one option)	Hair loss	645 (56.3)
	Acne	871 (76.1)
	Itching and redness of the skin	851 (74.3)
	Underarm darkening	144 (12.6)
	Inflammation and infection	55 (4.8)
From where did you buy the cosmetic product that caused your side effects?	Pharmacy	296 (25.8)
	Supermarkets	627 (54.6)
	Online stores	226 (19.6)

### Utilization patterns of cosmetic use

In terms of utilization patterns among participants who experienced side effects, 62.6% reported using the product weekly. The primary factors influencing cosmetic product selection were seller promotions (45.6%), followed by recommendations from others (22.9%). Notably, most participants (72%) did not read the leaflet accompanying the product.

Upon experiencing side effects, 58.1% discontinued the product within 2 to 3 days. The majority (83%) did not reuse the product after adverse reactions, and 88.3% did not seek specific treatment for the side effects (Table 3).

**Table 3:** Utilization patterns of cosmetic use

Utilization patterns		N (%)
How many times did you use the product that caused the side effects?	Daily	338 (29.4)
	Weekly	719 (62.6)
	Monthly	92 (8)
What are the criteria for choosing a cosmetic product?	Trademark	171 (14.9)
	Seller promotion	524 (45.6)
	Experiences of others	263 (22.9)
	Ingredients of the preparation	98 (8.5)
	Social media advertising	93 (8.1)
Did you read the leaflet accompanying the product?	Yes	322 (28)
	No	827 (72)
When did you stop using the cosmetic when side effects appeared?	Less than a day	145 (12.6)
	A day	90 (7.8)
	2–3 days	667 (58.1)
	4 days or more	247 (21.5)
Did you reuse the product after emergence of side effects	Yes	195 (17)
	No	954 (83)
Did you use specific treatment after emergence of side effects	Yes	134 (11.7)
	No	1015 (88.3)

### Association between cosmetic utilization and different age groups

A significant association was found between cosmetic utilization and different age groups (*P* < .05). Younger participants (18–22 years) were more likely to purchase cosmetics from pharmacies and use them daily. They preferred products based on brand reputation, read the accompanying leaflet, and discontinued use within a day if side effects appeared. They were also more likely to reuse the product and seek specific treatment for adverse reactions. Participants aged 23 to 30 years mostly bought cosmetics from supermarkets, used them monthly, and selected products based on seller promotions. They rarely read the leaflet and typically stopped using the product within 2 to 3 days when experiencing side effects. They were less likely to reuse the product or seek treatment. Participants over 30 years displayed distinct patterns of cosmetic utilization, as detailed in Table 4.

**Table 4:** Association between cosmetic utilization and different age groups

Variable	18–22 years N = 452	23–30 years N = 610	More than 30 years N = 83	*P* value
From where did you buy the cosmetic product that caused your side effects?				
Pharmacy	187 (41.4)	61 (10)	47 (56.6)	.001
Supermarkets	105 (23.2)	508 (83.3)	12 (14.5)	
Online stores	160 (35.4)	41 (6.7)	24 (28.9)	
How many times did you use the product that caused the side effects?				
Daily	223 (49.3)	62 (10.2)	52 (62.7)	.001
Weekly	166 (36.8)	13 (2.1)	18 (21.6)	
Monthly	63 (13.9)	535 (87.7)	13 (15.7)	
What are the criteria for choosing a cosmetic product?				
Trademark	108 (23.9)	34 (5.6)	28 (33.7)	.001
Seller promotion	22 (4.9)	496 (81.3)	4 (4.8)	
Experiences of others	195 (43.1)	42 (6.9)	25 (30.1)	
Ingredients of the preparation	67 (14.8)	19 (3.1)	12 (14.5)	
Social media advertising	60 (13.3)	19 (3.1)	14 (16.9)	
Did you read the leaflet accompanying the product?				
Yes	203 (44.9)	58 (9.5)	58 (69.9)	.001
No	249 (55.1)	525 (90.5)	25 (30.1)	
When did you stop using cosmetics when side effects appeared?				
Less than a day	104 (23)	22 (3.5)	18 (21.7)	.001
A day	62 (13.8)	9 (1.5)	19 (22.9)	
2–3 days	119 (26.3)	528 (86.6)	17 (20.5)	
4 days or more	167 (36.9)	51 (8.4)	29 (34.9)	
Did you reuse the product after emergence of side effects				
Yes	141 (31.2)	38 (6.2)	15 (18.1)	.001
No	311 (68.8)	572 (93.8)	68 (81.9)	
Did you use specific treatment after emergence of side effects				
Yes	85 (18.8)	25 (4.1)	23 (27.7)	.001
No	367 (81.2)	585 (95.9)	60 (72.3)	

* *P* value less than .05 is statistically significant.

## Discussion

### Participants sociodemographic

This study included 1,149 Saudi female respondents aged 18 years or older, with an average age of 25.3 ± 5.8 years. Understanding population characteristics is essential for assessing reported side effects and trends in cosmetic consumption. Our findings align with those of Shaaban et al., who reported higher cosmetic usage among younger females. This trend may be influenced by the younger generation’s increased engagement with social media platforms, which play a significant role in shaping beauty standards and consumer behavior.^18^ Additionally, the geographical distribution showed a higher population density in urban areas. This could be attributed to various factors, including the location of data collectors, which may have influenced participant recruitment.

### Cosmetic product use and side effects

Our findings indicated that face creams were the cosmetic products most frequently associated with side effects, reported by 73.4% of users, followed by makeup products (70.2%) and hair care products (59.1%). These results contrast with a previous study conducted in Saudi Arabia, where lotions were the most commonly reported product causing side effects (51.2%), followed by face creams (27.1%).^18^ The discrepancy in findings may be attributed to differences in utilization frequency within similar populations, potentially influenced by variations in study periods and the methods used for data collection and survey distribution among Saudi women.

The study results revealed that acne was the most frequently reported side effect (76.1%), followed by skin redness and itching (74.3%) and hair loss (56.3%). The least reported side effects were underarm darkening (12.6%) and inflammation or infection (4.8%). In contrast, the incidence of acne as a cosmetic-related side effect was significantly lower in other studies, with Udayanga et al.^2^ in Sri Lanka reporting 21.0% and Bilal et al.^1^ in Eastern Ethiopia reporting 16%. Variations in reported side effects may be attributed to demographic, cultural, and environmental factors, as well as differences in product formulations and usage patterns. These findings emphasize the importance of considering local contexts and population characteristics when evaluating cosmetic-related adverse effects.^16^

The primary reason for cosmetic use among participants was to enhance physical appearance (66.1%), a trend consistent with a study in Sri Lanka, where 89.3% of respondents cited the same motivation.^2^ Additionally, the majority of participants (54.6%) purchased their cosmetics from supermarkets, closely aligning with findings from Ethiopia, where 50.89% of participants reported the same purchasing preference.^3^ However, studies from other countries have indicated that pharmacies are the preferred choice for purchasing cosmetics.^19,20^

### Patterns of utilization

The frequency of cosmetic use significantly impacts the incidence of side effects. Previous research reported side effects in 42.3% of daily users and 8.8% of weekly users.^2^ Similarly, Getachew et al.^21^ found that 20% of daily cosmetic users experienced adverse reactions. In contrast, our study observed side effects in 29.4% of daily users, 62.6% of weekly users, and 8% of monthly users, suggesting a higher prevalence of irritants or increased sensitivity among weekly users. These findings emphasize the need for safer cosmetic formulations and greater consumer awareness regarding proper usage practices. The criteria for selecting cosmetic products also play a crucial role in consumer behavior and potential side effects. Our study found that 14.9% of participants based their choices on brand reputation, 45.6% on seller promotions, 22.9% on recommendations from others, 8.5% on ingredient composition, and 8.1% on social media advertising. These findings align with existing literature, which highlights the significant influence of marketing and peer recommendations on cosmetic product choices. Addis et al.^3^ reported that selecting products based on verified health claims was associated with a 97% lower likelihood of experiencing cosmetic-related adverse reactions. This underscores the importance of prioritizing ingredient transparency and certified health claims when choosing cosmetic products.

Additionally, Udayanga et al. found that 68.7% of consumers consider brand reputation a crucial factor in their purchasing decisions. In comparison, our study revealed that only 14.9% of participants selected products based on brand name, suggesting that brand reputation exerts a stronger influence in other markets.^2^ Reading the leaflet accompanying cosmetic products is essential for understanding proper usage and potential side effects. However, our study found that only 28% of participants read the leaflet, while 72% did not. This percentage is lower than that reported by Udayanga et al.,^2^ where 40.4% of consumers read ingredient labels and 45.4% reviewed labels for usage instructions. These findings highlight the need for increased consumer awareness regarding product information and safety. Several other studies have reported similar findings, indicating that reading user instructions, brand names, ingredient lists, expiration dates, and special remarks are common practices among cosmetic users.^22^ However, the low rate of leaflet reading in our study underscores the need for greater consumer education to promote informed usage and minimize the risk of adverse effects.

### Association between cosmetic utilization and different age groups

The association between cosmetic utilization and different age groups revealed significant differences across all variables (*P* < .05). Specifically, participants aged 18 to 22 demonstrated a greater tendency to purchase cosmetics from pharmacies (187; 41.4%) and use them daily (223; 49.3%). Brand reputation influenced their product selection (108; 23.9%), and when side effects occurred, they discontinued use within a day. However, they were more likely to resume using the product and seek specific treatment for adverse reactions. In contrast, a study examining cosmetic use and associated side effects among female university graduates reported that approximately half (172; 50.89%) purchased cosmetics from large supermarkets, while 61 (18.05%) preferred drug retail outlets. The study also found that 185 (54.73%) selected products based on brand name, aligning with our findings.^3^

Among participants aged 23 to 30, the majority purchased cosmetics from supermarkets (508; 83.3%), used them on a monthly basis (535; 87.7%), and primarily based their choices on seller promotions (496; 81.3%). When side effects emerged, they typically discontinued use within 2 to 3 days (528; 86.6%) and were less likely to reuse the product or seek specific treatment (572; 93.8%). The statistical differences in consumption habits across age groups suggest that younger women may be more aware of potential cosmetic-related side effects, even though they exhibit a higher tendency to resume product use after experiencing adverse reactions.

## Study limitations and future suggestions

Our study had several limitations. First, no objective clinical evaluation was conducted to confirm the reported adverse events. Participants provided responses based on their past experiences, making the study susceptible to recall bias. The findings may not be applicable to rural populations, as a significant portion of participants (82.7%) originated from urban settings. Additionally, the cross-sectional design prevented us from establishing a causal relationship between cosmetic use and adverse outcomes. It is crucial to carry out prospective cohort studies that observe the long-term impacts of cosmetic components, especially parabens and metal oxides, on both skin and overall health.^10^ Future research should incorporate clinical assessments to validate reported side effects, evaluate the effectiveness of specific treatments, and assess the impact of local campaigns and workshops in raising public awareness about cosmetic safety.

## Conclusion

This study highlighted the widespread use of cosmetics among Saudi women and identified significant side effects, including acne, redness, and hair loss. Most participants based their product choices on marketing rather than ingredient awareness, and a considerable number did not read product brochures, limiting informed decision-making. Younger women exhibited distinct purchasing and usage patterns, underscoring the need for targeted education on cosmetic safety. To enhance the safety of cosmetics, public health initiatives ought to concentrate on informing consumers about how to interpret labels, identify harmful substances, and report negative reactions. Awareness campaigns should prioritize younger audiences through social media platforms and university workshops to encourage informed choices and promote safer usage of cosmetic products.^19^ Despite these valuable insights, limitations such as recall bias and the absence of clinical evaluations suggest that further research is needed to investigate the long-term effects of cosmetic use.

### Recommendations for consumer education

To lessen the negative impacts of cosmetics, it is essential to implement focused educational strategies. Possible approaches include:

Initiating awareness campaigns via pharmacies and social media platforms.

Encouraging consumers to read product labels and ingredient lists carefully.

Organizing workshops at universities that address safe cosmetic practices.

Collaborating with dermatologists and influencers to disseminate reliable safety information.

These initiatives can enable women, particularly younger individuals, to make informed decisions and minimize preventable health risks.^16^

## Conflicts of interest

None.

## Funding

None.

## Study approval

The ethical approval (REC/SUR/BU-FM/2024/50) was obtained from Faculty of Medicine, Al-Baha University.

## Author contributions

All authors provide cooperations in the article production.
